# Onco-nephrology in clinical practice: pharmacokinetics, monitoring, and treatment strategies for patients with cancer and impaired renal function

**DOI:** 10.1007/s10147-025-02832-z

**Published:** 2025-07-17

**Authors:** Shunsaku Nakagawa, Keiko Ikuta, Takashi Masuda, Tomohiro Terada

**Affiliations:** Department of Clinical Pharmacology and Therapeutics, Kyoto University Hospital, 54 Shogoin-Kawahara-Cho, Sakyo-Ku, Kyoto, 606-8507 Japan

**Keywords:** Onco-nephrology, Pharmacokinetics, Dialysis, Proteinuria

## Abstract

Renal dysfunction is common in patients with cancer and affects their pharmacokinetics, thereby altering treatment efficacy and safety. This review outlines the principles of dose adjustment based on renal function and highlights specific issues in patients undergoing dialysis or with proteinuria. In patients undergoing dialysis, dose adjustment can be rational if drug properties such as molecular weight and protein binding are considered. Metabolites of some drugs, such as fluorouracil (5-FU), may accumulate in patients with impaired renal function, thereby increasing the risk of toxicity. For oxaliplatin, increased platinum exposure in patients undergoing dialysis does not necessarily increase toxicity, possibly because reactive platinum species are eliminated independent of renal function. Proteinuria can lead to reduced drug exposure to monoclonal antibodies owing to abnormal urinary excretion. The early detection and management of drug-induced kidney injuries are essential. These strategies include identifying risk factors, adjusting doses, and implementing monitoring systems. Protocol-based approaches, such as pharmacist-led monitoring, can improve cancer pharmacotherapy. Automated systems and AI-based models have also been explored for risk prediction. Future studies should focus on deepening our understanding of pharmacokinetics in patients with advanced chronic kidney disease (CKD) or those on dialysis. Multidisciplinary collaboration in onco-nephrology is important to improve cancer care in this growing population.

## Introduction

Patients with cancer often have multiple comorbidities, among which renal dysfunction is the most common. Twelve to twenty-five percent of all patients with cancer have chronic kidney disease (CKD) of stage 3 or higher (estimated glomerular filtration rate (eGFR) < 60 mL/min/1.73 m^2^) at the time of cancer diagnosis [1–3]. Furthermore, the risk of acute kidney injury (AKI) increases during cancer treatment, the perioperative period, and episodes of infection. In hospitalized patients, AKI occurs in approximately 5–30% of cases, with even higher rates observed in those requiring intensive care unit (ICU) management or undergoing procedures involving contrast media [4, 5]. AKI can be directly induced by nephrotoxic anticancer drugs such as cisplatin, methotrexate, and ifosfamide, which cause acute tubular necrosis and interstitial nephritis through direct injury to proximal tubular epithelial cells [6]. AKI has also been recognized as an immune-related adverse event (irAE) associated with immune checkpoint inhibitors (ICIs), occurring in approximately 2–5% of patients [7–9]. Renal dysfunction in patients with cancer is closely associated with limited treatment options and poor prognosis, highlighting the importance of timely and accurate assessment and appropriate management. Renal function significantly affects the pharmacokinetics of anticancer drugs. Therefore, when treating patients with impaired renal function, individualized dose adjustments and careful monitoring for adverse effects are essential.

In this review, we outline the principles of dose adjustment based on renal function. We also discuss newly recognized factors related to the pharmacokinetics of anticancer drugs in patients undergoing dialysis as well as those with proteinuria, practical approaches to the prevention and management of drug-induced kidney injury, and future directions for research.

## The basics of adjusting drug dosages based on renal function

Pharmacokinetics consists of four key processes: absorption, distribution, metabolism, and excretion. The kidneys play a central role in drug excretion from the body. Renal excretion is regulated by three mechanisms: glomerular filtration, tubular secretion, and tubular reabsorption. The impairment of renal function disrupts these processes, leading to prolonged drug retention. Therefore, to ensure safe and effective treatment, accurately assessing renal function before initiating therapy and tailoring drug selection and dosing are essential. Moreover, in cases of AKI, pharmacokinetics can change rapidly over a period of days to weeks. Continuous monitoring of renal function is crucial to appropriately respond to such fluctuations.

To estimate the clearance of renally excreted drugs in individual patients, the creatinine clearance (Ccr) and eGFR of each patient are typically calculated, compared with normal values, and extrapolated to predict drug clearance. The rationale for using these parameters is based on two main points: most renally excreted drugs are eliminated mainly through glomerular filtration, and creatinine serves as an endogenous marker that reflects renal function. Creatinine is generated from creatine in muscles at a relatively constant rate [10]. Once released into the blood, creatinine is filtered mainly through the glomeruli in the kidneys and excreted in the urine. This process is minimally affected by hepatic metabolism or intestinal excretion, and has a low protein-binding capacity, making creatinine a marker for estimating GFR. When renal function declines, creatinine excretion decreases, and serum concentration increases. Based on this inverse relationship, a variety of formulas have been developed to estimate GFR from serum creatinine levels. Representative equations include the Cockcroft–Gault formula, Modification of Diet in Renal Disease (MDRD) equation, and Chronic Kidney Disease Epidemiology Collaboration (CKD-EPI) equation [11]. More recently, eGFR equations specifically tailored to the Japanese population have been developed [12]. However, because creatinine production depends on muscle mass, GFR may be overestimated in elderly or underweight individuals. When necessary, additional biomarkers such as cystatin C should be considered [13].

**Fig. 1:**
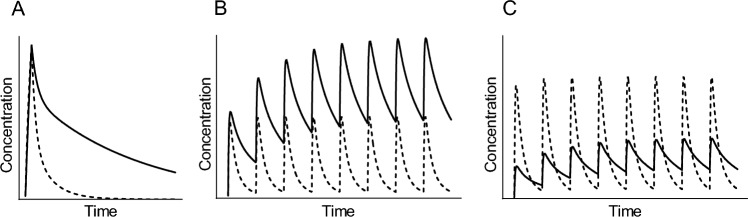
Pharmacokinetic profiles of patients with impaired renal function. We assume that a drug, whose total body clearance is approximately equivalent to renal clearance, is administered intravenously to two patients who differ only in renal function—one with normal kidney function (solid lines) and the other with impaired (dashed lines). **A** In single-dose administration, the maximum concentration (Cmax) may not change significantly, but the subsequent decline in concentration is slowed, leading to prolonged drug retention in the body. **B** This issue becomes particularly pronounced with repeated dosing, where drug accumulation can lead to excessive exposure, including an increase in Cmax. **C** Following the Giusti–Hayton method, adjusting the dose to reflect impaired renal function tends to result in a lower Cmax and higher trough concentrations

When renal function declines, drug excretion is delayed, resulting in a reduced rate of drug elimination from the body. Consequently, at the same dosage, the blood concentration of the drug is more likely to increase. In a single-dose administration, the maximum concentration (Cmax) may not change significantly; however, the subsequent decline in concentration is slowed, leading to prolonged drug retention in the body (Fig. 1A). This delay increases the area under the concentration–time curve (AUC), which may enhance therapeutic effects but also increase the risk of adverse reactions. This issue becomes particularly pronounced with repeated dosing, in which drug accumulation can lead to excessive exposure, including an increase in Cmax (Fig. 1B). Therefore, continuing the same dose in patients with impaired renal function as in those with normal renal function may result in excessively high blood concentrations and a significantly elevated risk of toxicity. To reduce this risk, two general dose adjustment strategies are commonly recommended, either alone or in combination: (1) extending the dosing interval to allow sufficient drug elimination before the next dose or (2) reducing the dose per administration to suppress the Cmax.

One specific method for adjusting drug dosage based on renal function is the Giusti–Hayton method [14]. This mathematical model could be used to modify the dosage or dosing intervals of renally excreted drugs. In this method, the correction factor (G) is defined as follows:

$$G = {1} - \left( {\text{fraction excreted unchanged in urine}} \right) \times ({1} - {\text{fraction ofretained renal function}})$$

Using this *G* value, the dosing regimen can be adjusted in one of the following ways: (1) dividing the standard dosing interval by *G* to determine the appropriate interval based on renal function; (2) multiplying the standard dose by *G* to calculate an adjusted dose per administration; or (3) multiplying the dose-to-interval ratio (standard dose/standard interval) by *G* and adjusting both the dose and interval accordingly. Flexible dosing adjustments can be achieved using this method. However, when such dose or interval adjustments are made, although the average blood concentration and AUC may be maintained, the pharmacokinetic profile differs from that in patients with normal renal function; the Cmax tends to be lower and the trough concentration tends to be higher (Fig. 1C). Evidence-based dosing strategies have been established for several anticancer drugs for patients with impaired renal function [15]. Among these, carboplatin is commonly administered using the Calvert formula, a well-established method that individualizes dosage based on renal function and conceptually resembles the Giusti–Hayton method by linking the target AUC to the patient’s GFR. In the case of cisplatin, a current guideline recommend stepwise dose reductions or interval adjustments based on eGFR, typically reducing the dose by 25–50% when eGFR falls below 60 mL/min/1.73 m^2^ and avoiding administration when it drops below 45 mL/min/1.73 m^2^ [15].

## Pharmacokinetics of anticancer drugs in patients undergoing dialysis

The number of patients undergoing dialysis and receiving cancer pharmacotherapy is increasing. A multicenter retrospective study conducted in Japan reported that the median overall survival of patients receiving palliative chemotherapy was 13 months, whereas the 3-year survival rate of patients receiving perioperative chemotherapy was 79% [16]. This finding suggests that with appropriate assessment of the patient’s condition and dosage adjustment, effective and safe cancer pharmacotherapy may be feasible, even in patients undergoing dialysis.

In patients undergoing dialysis, although renal function is markedly impaired, hepatic and other physiological functions are not necessarily compromised. Therefore, designing rational dosing regimens for this population is often feasible. In particular, understanding whether a drug is removed by dialysis is useful for determining appropriate dose adjustments. The extent to which a drug is cleared by dialysis depends on its molecular weight, water/lipid solubility, protein binding, and volume of distribution [17, 18]. In general, drugs with a low molecular weight, high water solubility, and low protein binding are more likely to be removed by dialysis. Conversely, lipophilic drugs typically possess a large distribution volume and high tissue permeability; therefore, they are less likely to be removed from the blood, and the efficacy of dialysis in clearing such drugs is limited. For example, among the platinum-based drugs, cisplatin has a large distribution volume and high tissue penetration; therefore, its removal by dialysis is minimal [19]. In contrast, carboplatin has a relatively smaller volume of distribution and is partially removed by dialysis [20]. Furthermore, the drug clearance per unit time achieved by dialysis may exceed the patient’s native renal clearance. As a result, blood concentrations may decrease rapidly during dialysis, followed by a rebound increase owing to redistribution from peripheral tissues into the blood.

**Fig. 2:**
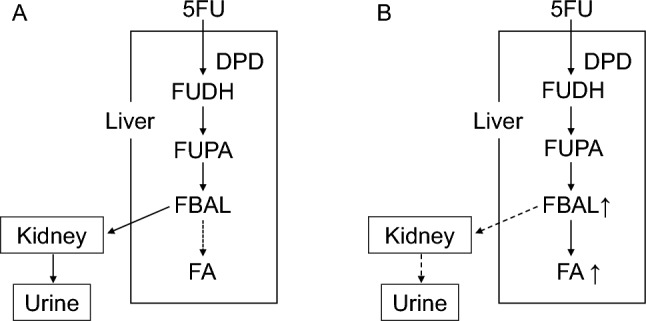
Metabolic pathways of 5-fluorouracil (5-FU). **A** 5-FU is mainly metabolized in the liver by dihydropyrimidine dehydrogenase (DPD), initially converting to dihydrofluorouracil (FUDH), then to fluoro-ureidopropionic acid (FUPA), and subsequently to FBAL. FBAL is eliminated by the kidneys but can be further metabolized into fluoroacetate (FA). Over 80% of administered 5-FU undergoes this metabolic pathway. **B** In patients with significantly impaired renal function, renal clearance of FBAL would be significantly decreased, and caution is required regarding the accumulation of FBAL or FA

On the other hand, even drugs for which the kidneys do not play a major role in metabolism or inactivation may exhibit pharmacokinetic profiles that differ from those in patients with normal renal function. 5-Fluorouracil (5-FU) is primarily eliminated by the liver; thus, renal function is considered to have little effect on its pharmacokinetics or efficacy (Fig. 2). However, recent reports have shown that in patients with impaired renal function, especially those undergoing dialysis, the metabolites of 5-FU, fluoro-β-alanine (FBAL) and fluoroacetate (FA), can accumulate after administration [21]. Furthermore, patients undergoing dialysis may have an increased risk of hyperammonemia associated with 5-FU treatment [22–24]. The toxicity associated with these metabolites is thought to occur via mechanisms distinct from those of 5-FU. 5-FU is mainly metabolized by dihydropyrimidine dehydrogenase, including an initial conversion to dihydrofluorouracil, then to fluoroureidopropionic acid, and subsequently to FBAL. FBAL is further metabolized into compounds such as FA, and some of these water-soluble metabolites can be eliminated by the kidneys (Fig. 2A). It is estimated that over 80% of administered 5-FU undergoes this metabolic pathway [21]. Therefore, attention should be paid to the potential accumulation of these metabolites in patients with significantly impaired renal function (Fig. 2B). Recent reports have suggested that adjusting the timing of dialysis may help reduce metabolite accumulation and prevent adverse effects. For example, when 5-FU is administered as a 48 h continuous infusion, dialysis on the day following the start of administration may help reduce the accumulation of 5-FU metabolites and the risk of hyperammonemia [23].

**Fig. 3:**
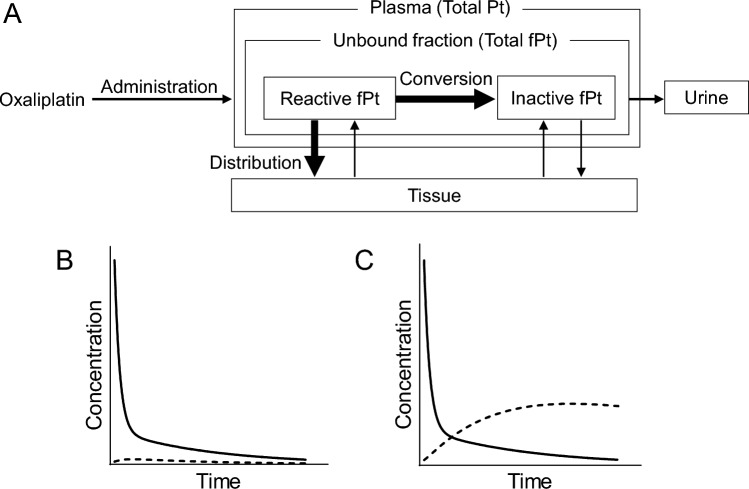
Pharmacokinetic profiles of oxaliplatin in patients undergoing dialysis. **A** In patients administered oxaliplatin, plasma ultrafilterable platinum (fPt) consists of a mixture of reactive platinum species capable of binding to biological molecules, such as DNA, and inactive platinum species that have lost their reactivity. The decline in the plasma concentration of reactive fPt is thought to occur primarily through a non-enzymatic conversion to inactive species or through distribution into peripheral tissues. **B, C** Assuming a drug like oxaliplatin, for which the kidneys play only a minor role in metabolism or inactivation, but whose metabolites are primarily excreted renally, changes in blood concentrations following a single dose can be predicted. The solid line represents the parent compound, while the dashed line represents the metabolite. Compared to a patient with normal renal function (**B**), a patient with impaired renal function (**C**) is expected to show a gradual increase in blood levels of the metabolite after administration. A similar trend is expected not only with bolus injection, but also during constant-rate intravenous infusion

Because renal excretion is a major pathway for eliminating platinum, Ccr and the clearance of plasma ultrafilterable platinum (fPt) are significantly correlated in patients administered oxaliplatin. Thus, when a constant dose is administered, regardless of renal function, the AUC of fPt increases with decreasing renal function. However, this increase in fPt is not correlated with the incidence or severity of adverse events [25]. One possible explanation for these results is that fPt in the plasma consists of a mixture of reactive platinum species capable of binding to biological molecule, such as DNA and inactive platinum species that have lost their reactivity [26]. Therefore, renal function may have a limited effect on the elimination of pharmacologically reactive platinum (Fig. 3A). In our study using an animal model, we found that plasma concentrations of DNA-reactive platinum declined rapidly after oxaliplatin administration, regardless of renal function [27]. Moreover, in a patient undergoing dialysis with advanced gastric cancer treated with mFOLFOX7, total platinum and fPt concentrations decreased to approximately 35.9% and 7.3%, respectively, on days 2 and 14, respectively, post administration. In contrast, the DNA-reactive fPt had already decreased to 1.9% and 0.6%, respectively, at the same time points, indicating more rapid elimination. These findings suggest that pharmacologically reactive platinum, which is responsible for both therapeutic efficacy and toxicity, may be rapidly cleared from the body, regardless of renal function. Indeed, although high levels of platinum exposure have been observed in patients undergoing dialysis and receiving oxaliplatin, the treatment is generally well-tolerated [28, 29]. These findings underscore the importance of evaluating not only the pharmacokinetics of the parent drug, but also those of its metabolites, including their accumulation and potential bioactivity, when optimizing pharmacotherapy in this population (Fig. 3B, C).

## Proteinuria and pharmacokinetics

Proteinuria refers to the presence of proteins in the urine resulting from impaired selective permeability of the glomerular filtration barrier. Inhibitors of vascular endothelial growth factor (VEGF) or its receptor, such as bevacizumab and ramucirumab, cause proteinuria via VEGF signal inhibition and renal endothelial cell or podocyte dysfunction [30]. Traditionally, proteinuria has been widely used as a marker of glomerular or tubular injury in kidney disease. However, recent studies have shown its potential impact on pharmacokinetics, particularly of drugs with high molecular weights or protein-binding capacities [31, 32].

Monoclonal antibody drugs such as bevacizumab and nivolumab have large molecular weights and are generally considered poorly eliminated by the kidneys. However, in cases where the glomerular filtration barrier is disrupted, these high-molecular-weight agents may be abnormally excreted into the urine, potentially resulting in decreased serum concentrations [31]. In patients with a urinary protein-to-creatinine ratio (UPCR) ≥ 1 g/g, dose-adjusted trough concentrations of bevacizumab and nivolumab in plasma were significantly reduced compared to those in patients with lower UPCRs. Furthermore, a separate cohort study developed a population pharmacokinetic model for bevacizumab, identifying UPCR as a significant covariate influencing drug clearance [32]. Simulations based on this model showed that trough concentrations in the plasma could decrease by up to 30%, depending on the severity of proteinuria. These results indicate that proteinuria may contribute to increased drug clearance and reduced systemic exposure to monoclonal antibodies. Thus, proteinuria is not only a marker of renal dysfunction but also a clinically relevant variable that may influence the pharmacokinetics and therapeutic efficacy of antibodies. However, whether the increased clearance of antibodies directly translates to reduced clinical efficacy is unclear. Whether a decrease in trough concentration leads to a diminished therapeutic effect or whether maintaining a certain level of drug exposure is sufficient to preserve efficacy remains to be clarified. Further detailed pharmacokinetic and pharmacodynamic studies are required to address these issues.

## Management of drug-induced renal injury in patients with cancer: protocol- and automation-based approaches

Early recognition and proactive management of nephrotoxicity are vital to ensure the safety and efficacy of cancer pharmacotherapy. For the management of drug-induced kidney injury, clinicians should assess the presence of underlying conditions such as CKD, diabetes, and hypertension, as well as additional risk factors such as dehydration, infection, prior use of contrast media, and concomitant use of potentially nephrotoxic drugs. Renal function can be estimated using eGFR formulae. The implementation of drug-specific preventive measures is also important. For example, adequate hydration is recommended before and after administration [33]. Therefore, regular monitoring of proteinuria using anti-VEGF inhibitors is essential. For anti-EGFR antibodies, such as cetuximab and panitumumab, periodic measurement of serum magnesium levels and supplementation as needed are important for the prevention of hypomagnesemia. In patients receiving ICIs, irAEs may affect multiple organs, including the kidneys; therefore, comprehensive monitoring is required. Therefore, effective prevention and early detection of drug-induced kidney injury require drug-specific risk assessment and careful scheduling of laboratory tests. A clinical system should be implemented to ensure that the necessary tests are performed at the right time and that any abnormalities are managed without delay.

Effective early detection and management of drug reactions require a multidisciplinary team comprising physicians, pharmacists, nurses, and other essential healthcare providers. In the context of ICIs, the effectiveness of protocol-based monitoring systems has been demonstrated. For example, in a study involving patients with lung cancer, the introduction of pharmacist-led Protocol-Based Pharmacotherapy Management (PBPM) significantly improved the rate of laboratory tests for irAEs [34]. In this protocol, both the type and timing of tests are predefined, and pharmacists are responsible for reviewing and supplementing test orders issued by physicians. This system helps to prevent missed laboratory assessments and contributes to improved treatment safety.

Algorithm-based and automated approaches are also useful for the early detection of kidney injury. Clinical decision support systems (CDSS) integrated into electronic health records that generate automated alerts when renal function indicators exceed predefined thresholds are useful. For instance, a multicenter study evaluated the effect of CDSS implementation on AKI [35]. Analysis of data from over 528,000 patients revealed that the implementation significantly reduced hospital mortality, dialysis initiation, and length of hospital stay among patients with AKI. These findings suggest that integrating AKI detection algorithms into CDSS can improve patient outcomes by facilitating early recognition and timely management of AKI. In addition, recent advances have been made in the development of AI-based models to predict the risk of kidney injury [36, 37]. These models use multivariate patient data, including laboratory values, concomitant medications, and medical history, to predict AKI onset through machine learning techniques. The integration of such models into CDSS may enable the earlier identification of optimal intervention timing and promote personalized medical care.

## Future perspectives

Evidence of the pharmacokinetics of anticancer drugs in patients with end-stage renal disease remains limited, and drug-specific strategies for optimal dosing and assessment of drug clearance are still required. For patients undergoing dialysis, treatment planning must consider not only dialyzability and the timing of dialysis, but also the accumulation of metabolites and the associated risk of toxicity.

The development of biomarkers and monitoring techniques that enable early detection of pharmacokinetic changes is essential. Although biomarkers such as neutrophil gelatinase-associated lipocalin (NGAL) and kidney injury molecule-1 (KIM-1) have attracted attention as indicators of AKI [38], the need to identify novel markers that reflect cancer treatment-related kidney injury is growing. The integration of such biomarkers with therapeutic drug monitoring (TDM) and pharmacokinetic modeling enables the visualization of time-dependent pharmacokinetic changes and supports individualized dose optimization.

Renal dysfunction may alter drug clearance and the volume of drug distribution through mechanisms such as proteinuria and hypoalbuminemia. Although these effects are not yet fully understood or adequately addressed in clinical practice, they are particularly important for biologics with large molecular weights, such as monoclonal antibodies. For these drugs, pharmacokinetic modeling must consider factors such as molecular size, endogenous recycling pathways, tissue distribution, and redistribution.

Future clinical trials should include patients with advanced CKD or those undergoing dialysis, and treatment protocols should be continually revised based on pharmacokinetic parameters. Addressing these challenges requires strengthening the interdisciplinary collaboration in the field of onco-nephrology.

## Data Availability

As this is a review article, no new data were generated or analyzed in this study. Thus, data sharing is not applicable.
